# Statins decrease leptin expression in human white adipocytes

**DOI:** 10.14814/phy2.13566

**Published:** 2018-01-26

**Authors:** Prachi Singh, Yuebo Zhang, Pragya Sharma, Naima Covassin, Filip Soucek, Paul A. Friedman, Virend K. Somers

**Affiliations:** ^1^ Department of Cardiovascular Medicine Mayo Clinic Rochester Minnesota; ^2^ ICRC ‐ Department of Cardiovascular Diseases St. Anne's University Hospital, Brno Czech Republic

**Keywords:** Leptin, satiety, statins

## Abstract

Statin use is associated with increased calorie intake and consequent weight gain. It is speculated that statin‐dependent improvements in lipid profile may undermine the perceived need to follow lipid‐lowering and other dietary recommendations leading consequently to increased calorie intake. However, increases in calorie intake in statin users may also be related to statin‐dependent decreases in satiety factors such as leptin, an adipocyte‐derived adipokine. The objective of our study was to examine the direct effects of statins on leptin expression. Adipocytes are the main source of circulating leptin. Therefore, we examined the effects of atorvastatin and simvastatin on leptin expression in cultured human white adipocytes. We show that treatment of white adipocytes with simvastatin and atorvastatin decreases leptin mRNA expression (simvastatin: *P* = 0.008, atorvastatin: *P* = 0.03) and leptin secretion (simvastatin: *P* = 0.0001, atorvastatin: *P* = 0.0001). Both simvastatin and atorvastatin mediate decreases in leptin expression via extracellular‐signal‐regulated kinases 1/2 and peroxisome proliferator‐activated receptor gamma pathways (simvastatin: *P* = 0.01, atorvastatin: *P* = 0.026). Additionally, statin treatment also induced expected increases in adiponectin, while decreasing monocyte chemoattractant protein 1 (MCP1) mRNA. Furthermore, statins increased secretion of both total as well as high molecular weight adiponectin while decreasing MCP1 secretion. To conclude, statins act directly on human white adipocytes to regulate adipokine secretion and decrease leptin expression. Leptin is an important satiety factor. Hence, statin‐dependent decreases in leptin may contribute, at least in part, to increases in food intake in statin users.

## Introduction

Dyslipidemia is among the most common risk factors contributing to the development of cardiovascular disease. The American Heart Association/American College of Cardiology (AHA/ACC) 2013 guidelines for the treatment of blood cholesterol to reduce atherosclerotic cardiovascular risk in adults recommends life style modifications prior to, and in concert with, use of moderate‐to‐high intensity statins to achieve lipid‐lowering goals (Stone et al. 2014). Similarly, the European Atherosclerosis Society and the European Society of Cardiology (EAS/ESC 2011) guidelines also recommend life style interventions along with lipid‐lowering drugs to achieve reduced lipid levels (Reiner et al. 2011; Zoungas et al. 2014). Interestingly, statin users in the general US population (as represented in the National Health and Nutrition Examination Survey, NHANES) report higher total calorie intake as well higher fat intake compared to nonstatin users (Sugiyama et al. 2014). Additionally, the average daily calorie intake among individuals undergoing statin therapy in the 2009–2010 period was found to be 9.6% higher than those in the 1999–2000 period. The increased calorie consumption in statin users has been attributed to a sense of false assurance provided by remarkable improvements in lipid levels, resulting in less stringent dietary control.(Athyros and Mikhailidis 2014; Redberg 2014; Sugiyama et al. 2014).

Leptin, an adipokine, is an important satiety‐inducing factor in appetite regulation (Friedman and Halaas 1998; Elmquist et al. 2005). Increases and decreases in leptin cause decreases and increases in food intake, respectively (Ahima et al. 1996). Indeed, leptin deficiency in humans is associated with hyperphagia and severe obesity, and treatment with leptin reduces food intake and consequently causes weight loss (Montague et al. 1997; Farooqi et al. 2002). Furthermore, a disproportionate decrease in leptin during weight loss is linked to increased hunger and weight regain after initial weight loss (Sumithran et al. 2011). Exogenous leptin replacement during weight loss is associated with increased satiety and long‐term maintenance of weight loss (Rosenbaum et al. 2008; Kissileff et al. 2012). Considering the critical role of leptin in appetite regulation, we hypothesized that increased calorie intake in statin users may be secondary to statin‐mediated decreases in leptin. Therefore, we examined the role of statins in reducing leptin expression in cultured human white adipocytes and explored the potential mechanistic pathways mediating these effects. Since previous studies have consistently shown statin‐dependent decreases in monocyte chemoattractant protein 1 (MCP1) (Han et al. 2005; Abe et al. 2008; Lobo et al. 2012; Dworacka et al. 2014) and increases in adiponectin, (Wanders et al. 2010) we also examined the effects of statin on these adipokines. These measures served as a control of our experiments examining the effects on leptin.

## Methods

### Cell culture

In vitro experiments were done using commercially available primary human white preadipocytes (HWP) isolated from subcutaneous abdominal adipose tissue of lean and obese subjects (Zen‐Bio Inc, North Carolina). Cells were grown in subcutaneous preadipocyte growth media containing growth supplements and fetal bovine serum (Zen‐Bio) to confluence and differentiated in the presence of adipocyte differentiation media (Zen‐Bio) for 7 days followed by additional growth for 7 days in adipocyte maintenance media (Zen‐Bio). After the 14 days of initiation of differentiation, experiments were conducted following overnight incubation in basal media (Zen‐Bio) lacking supplements and serum.

### Statins and adipokines

To determine the effect of statins on leptin mRNA, differentiated HWP were treated with increasing concentrations of simvastatin (0–1 *μ*mol/L) (Sigma‐Aldrich, St. Louis) and atorvastatin (0–10 *μ*mol/L) (Sigma‐Aldrich, St. Louis) for 6 h. Atorvastatin stock (5 mg/mL) solution was made in DMSO. Control experiments for atorvastatin also contained appropriate volume of DMSO. Simvastatin stock (4 mg/mL) was made by dissolving 4 mg simvastatin in 100 *μ*L ethanol and 150 *μ*L sodium hydroxide (0.1 N), activated at 50°C for 2 h, the pH was adjusted to 7.0 by HCl, and the volume was made up to 1 mL using basal adipocyte media (Sadeghi et al. 2000). Simvastatin control was made similarly. The range of statin concentrations used for these experiments was determined based on published literature (Negre‐Aminou et al. 1997; Krysiak et al. 2009; Lobo et al. 2012). Total RNA was isolated using PureLink RNA isolation kit (Ambion) and cDNA library was created using high‐capacity cDNA reverse transcription kit (Applied Biosystems). Changes in leptin, adiponectin, and MCP1 mRNA with statin treatment were determined semi‐quantitatively using commercially available TaqMan probes as per manufacturer's instruction. GAPDH was used as an endogenous control for these experiments.

Similarly, effect of statins on leptin, MCP1, total adiponectin, and high molecular weight adiponectin secretion was determined in conditioned medium after 24‐h treatment using quantikine human ELISA kits as per manufacturer's instruction (RnD systems, Minneapolis).

To examine the role of ERK1/2 and PPAR*γ* pathways on statin‐dependent changes in leptin, MCP1, and adiponectin secretion, cells were incubated with PD98059 (30 *μ*mol/L, Sigma‐Aldrich, St. Louis) or T0070907 (10 *μ*mol/L, Sigma‐Aldrich, St. Louis) for 30 min before and during treatment with atorvastatin (10 *μ*mol/L) or simvastatin (1 *μ*mol/L). Conditioned media (24 h) from these experiments was analyzed for secreted leptin, MCP1, and adiponectin using quantikine ELISA kit (RnD systems, Minneapolis).

### Statistics

Data were analyzed using JMP 9.0.1 software (SAS Institute Inc.) and presented as mean ± SEM. All in vitro experiments were performed at least three independent times. Statistical significance and pairwise analysis were determined using Wilcoxon rank‐sum test.

## Results

**Figure 1:**
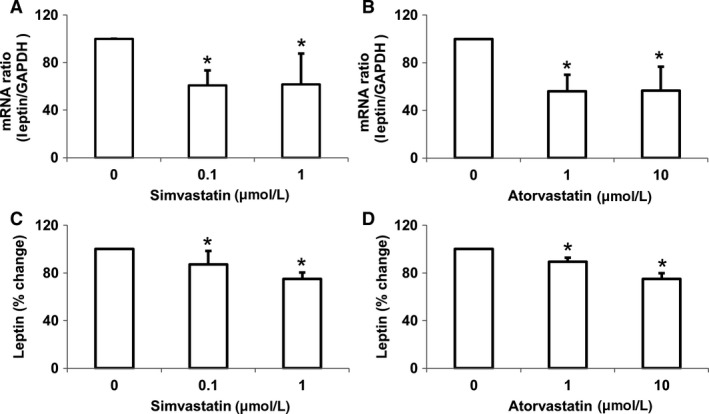
Statins reduce leptin expression in white adipocytes. Treatment with increasing concentrations of simvastatin lowered leptin mRNA (A) and leptin secretion (B). Treatment with increasing concentrations of atorvastatin lowered leptin mRNA (C) and leptin secretion (D). Data are presented as mean ± SEM (*n* = 3 independent experiments). *P <* *0.05 compared with control as determined by Wilcoxon rank‐sum test. Appropriate vehicle controls were used for each experiment.

**Figure 2:**
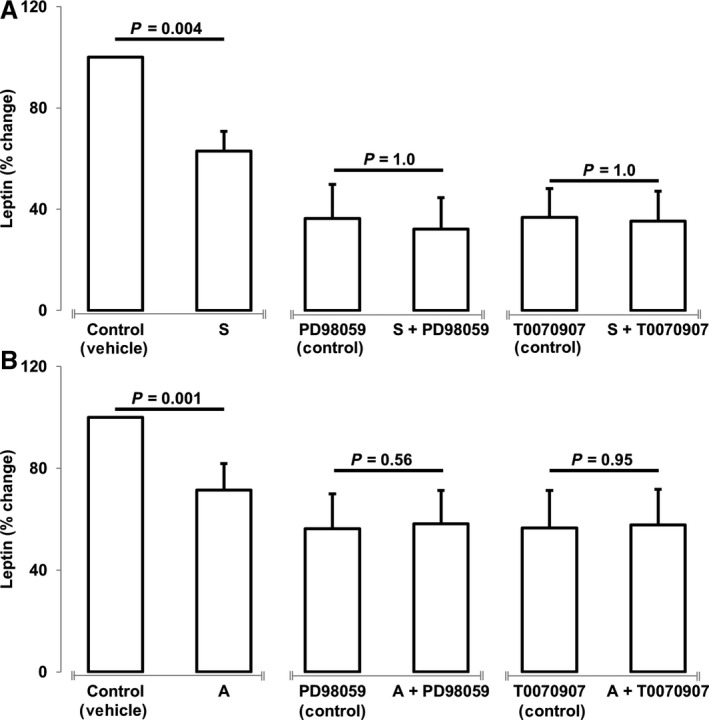
Statins mediate reductions in leptin expression via ERK1/2 and PPAR *γ* cellular signaling pathways. Treatment of human white adipocytes with statins in the presence of ERK1/2 upstream inhibitor (PD98059) and PPAR *γ* inhibitor (T0070907) prevented simvastatin (S, 1 *μ*mol/L) (A) and atorvastatin (A, 10 *μ*mol/L) (B) dependent decreases in leptin secretion. Data are presented as mean ± SEM (*n* = 3 independent experiments). *P <* *0.05 compared with control as determined by Wilcoxon rank‐sum test.

We examined the effects of simvastatin and atorvastatin on regulation of leptin using white adipocytes. Both simvastatin and atorvastatin decrease the expression of leptin mRNA (simvastatin: *P* = 0.008, atorvastatin: *P* = 0.03) as well as leptin secretion (simvastatin: *P* = 0.0001, atorvastatin: *P* = 0.0001) (Fig. 1). Furthermore, we examined if the effect of statins on leptin secretion is mediated via the activation of extracellular‐signal‐regulated kinases 1/2 (ERK1/2) and peroxisome proliferator‐activated receptor gamma (PPAR*γ*) pathways (Fig. 2). Inhibition of ERK1/2 activation via PD98059 and inhibition of PPAR*γ* via T0070907 prevented statin‐mediated decreases in leptin secretion (simvastatin: *P* = 0.002, atorvastatin: *P* = 0.001). Importantly, incubation of cells in the presence of ERK1/2 and PPAR*γ* reduced leptin secretion to the same level as that induced by atorvastatin and simvastatin.

**Figure 3:**
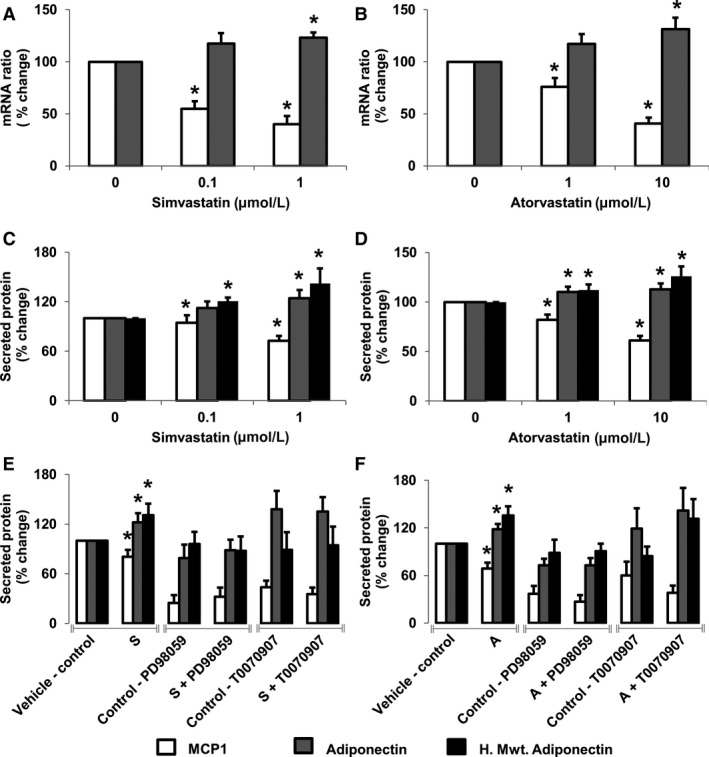
Statins reduce monocyte chemoattractant protein 1 (MCP1) while increasing adiponectin expression in white adipocytes. Treatment with increasing concentrations of simvastatin/atorvastatin decreased MCP1 and increased adiponectin mRNA (A/B) and protein secretion (C/D). Treatment with ERK1/2 upstream inhibitor (PD98059) and PPAR *γ* inhibitor (T0070907) prevented simvastatin (E) and atorvastatin (F) mediated changes in adipokines. Data are presented as mean ± SEM (*n* = 4 independent experiments). *P <* *0.05 compared with control as determined by Wilcoxon rank‐sum test. White bars (MCP1), gray bars (adiponectin), and black bars (high molecular weight adiponectin).

Additionally, we examined the effects of simvastatin and atorvastatin on expression and secretion of MCP1 and adiponectin. Treatment of white adipocytes with atorvastatin and simvastatin decrease MCP1 mRNA (simvastatin: *P* = 0.003, atorvastatin: *P* < 0.001) and MCP1 protein secretion (simvastatin: *P* < 0.001, atorvastatin: *P* < 0.001) while increasing adiponectin mRNA (simvastatin: *P* = 0.019, atorvastatin: *P* = 0.025) and adiponectin protein secretion (simvastatin: *P* = 0.0048, atorvastatin: *P* = 0.007) (Fig. 3). Importantly, simvastatin and atorvastatin‐mediated increases in adiponectin also increases high molecular weight adiponectin secretion (simvastatin: *P* = 0.012, atorvastatin: *P* = 0.005).

When compared to vehicle control for simvastatin, treatment of cells with ERK1/2 inhibitor (PD98059) and PPAR*γ* inhibitor (T0070907) decreased the secretion of MCP1 (*P* = 0.004, and *P* = 0.004, respectively) but had no effect on secretion of total adiponectin (*P* = 0.12 and *P* = 0.07, respectively). Also while ERK1/2 inhibition did not alter secretion of high molecular weight adiponectin (*P* = 0.1133), inhibition of PPAR*γ* marginally decreased high molecular weight adiponectin secretion (*P* = 0.007). When compared to vehicle control for atorvastatin, ERK1/2 inhibition decreased secretion of MCP1 (*P* = 0.004) and total adiponectin (*P* = 0.006) but had no effect on high molecular weight adiponectin secretion (*P* = 0.08). However, inhibition of PPAR*γ* pathway did not alter secretion of MCP1 (*P* = 0.40), adiponectin (*P* = 1.0), or high molecular weight adiponectin (*P* = 0.40).

Furthermore, addition of simvastatin or atorvastatin to cells in the presence of the ERK1/2 and PPAR*γ* inhibitors did not further alter secretion of MCP1 (PD98059 vs. PD98059+simvastatin, *P* = 0.52; T0070907 vs. T0070907+simvastatin, *P* = 0.49; PD98059 vs. PD98059+atorvastatin, *P* = 0.49; T0070907 vs. T0070907+atorvastatin; *P* = 0.43), adiponectin (PD98059 vs. PD98059+simvastatin, *P* = 0.68; T0070907 vs. T0070907+simvastatin, *P* = 0.93; PD98059 vs. PD98059+atorvastatin, *P* = 0.95; T0070907 vs. T0070907+atorvastatin, *P* = 0.37); or high molecular weight adiponectin (PD98059 vs. PD98059+simvastatin, *P* = 0.44; T0070907 vs. T0070907+simvastatin, *P* = 0.89; PD98059 vs. PD98059+atorvastatin: *P* = 0.32; T0070907 vs T0070907+atorvastatin, *P* = 0.10) (Fig. 3E and F). Together, these studies suggest that ERK1/2 and PPAR*γ* pathway are important for the statin‐mediated regulation of MCP1, total and high molecular weight adiponectin.

## Discussion

The role of statins in regulation of leptin is conflicting. While several clinical studies suggest that statin therapy is associated with decreased systemic leptin (Sun et al. 2010; Bellia et al. 2012; Buldak et al. 2012; Takahashi et al. 2012; Krysiak et al. 2014), some studies have shown that statin therapy does not contribute to any change in leptin levels (Chu et al. 2008; Szotowska et al. 2012; Al‐Azzam et al. 2013). These discrepancies may be related to differences in study populations, presence of comorbidities, dosage of statins, length of statin treatment, as well as use of different statins. Therefore, to directly determine the effect of statins on regulation of leptin in the absence of other confounding variables, we used an in vitro approach. To the best of our knowledge, we show for the first time that simvastatin and atorvastatin decrease the leptin expression in primary human adipocytes. These results are consistent with a previous in vitro study using mice 3T3‐L1 cells showing simvastatin‐dependent decreases in leptin (Maeda and Horiuchi 2009). However, our findings are in contrast to a previous ex vivo study which showed that atorvastatin treatment had no effect on leptin release (Krysiak et al. 2009). This discrepancy from the ex vivo study may be related to different approaches using in vitro cells versus ex vivo adipose tissue explants. Adipose tissue consists of several cell types including immune cells which may alter overall response to statins by contributing to a microenvironment different from adipocytes in controlled cell culture conditions. Importantly, the participants included diabetic and prediabetic individuals (indicated by mean HbA_1C_ > 5.9 in both groups) which would also suggest altered/impaired cellular signaling mechanisms. We used increasing concentrations of atorvastatin and also examined the effects of statins on leptin mRNA and leptin secretion. We also demonstrate the role of ERK1/2 and PPAR*γ* pathways in statin‐mediated regulation of leptin, MCP1, and adiponectin. Since previous studies have suggested that ERK acts through the activation of PPAR*γ* pathways to modulate transcription of target proteins (Paumelle and Staels 2007), it is likely that statins activate ERK1/2 which in turn activates PPAR*γ* and thereby decreases the transcription of leptin mRNA. Indeed, statins have been previously reported to increase PPAR*γ* activity via ERK1/2 activation to decrease inflammation in other cells such as monocytes and macrophages (Yano et al. 2007). Of note, we also show statin‐mediated decreases in MCP1 and increases in adiponectin. These findings are consistent with previous literature (Hu et al. 2009; Koh et al. 2011; Buldak et al. 2012; Lobo et al. 2012; Krysiak et al. 2014), and are concordant with the pleiotropic anti‐inflammatory effect of statins.

In the previous study by Maeda and Horiuchi (2009) simvastatin‐mediated decreases in leptin mRNA were shown to be dependent on cellular increases in cAMP and activation of the PKA pathway. The authors also state that inhibition of ERK1/2 pathway with PD98059 did not alter leptin transcription and proposed that this pathway may not be important for statin‐dependent lowering of leptin mRNA. However, key experiments examining the effects of ERK inhibition in the presence of simvastatin were not conducted. Therefore, it cannot be stated that activation of ERK1/2 pathway is not required for simvastatin‐mediated decreases in leptin. In contrast, we observed a sharp decrease in leptin secretion in the presence of ERK1/2 and PPAR*γ* inhibitors (Fig. 2A). Furthermore, in the presence of these inhibitors, neither simvastatin nor atorvastatin was able to alter leptin secretion. These results suggest that activation of ERK1/2 and PPAR*γ* is required for statin action. Alternatively, it is possible that inhibition of these pathways lowers the secretion of leptin such that a low threshold limit is reached and statins are unable to further reduce leptin secretion. Nonetheless, we clearly show that activation of ERK and PPAR*γ* are important to regulate leptin secretion. The discrepancies in underlying pathways may be related to differences in species and may also be indicative of a crosstalk between different cell signaling pathways. Indeed, studies have shown that PPAR activity is regulated by phosphorylation of several kinases including ERK1/2 and PKA (Burns and Vanden Heuvel 2007). Moreover, cAMP mediated activation of ERK1/2 via PKA has been demonstrated in some cell types (Belcheva and Coscia 2002). Therefore, it is likely that PKA activation may be a process upstream of ERK1/2 and PPAR*γ* activation for statin‐mediated regulation of leptin transcription and secretion.

Our study shows an approximate 20% reduction in leptin transcription in response to atorvastatin and simvastatin treatment. Atorvastatin use in obese individuals with type 2 diabetes has also been shown to cause up to 40% reduction in circulating leptin (von Eynatten et al. 2005). Notably, studies examining the changes in leptin with diet‐induced weight loss have shown an approximate 30–22% reduction in systemic leptin with 10–3% lowering of body weight (Rosenbaum et al. 2005; Shai et al. 2008). This weight loss and decreases in leptin were also accompanied by the expected increases in hunger, increased sensitivity to food cues, and decreases in satiety which are known to contribute to increased food intake and consequent difficulties associated with weight maintenance, with resulting weight regain (Rosenbaum et al. 2008; Hinkle et al. 2013). Interestingly, replenishing leptin to levels prior to weight loss intervention has been shown to reverse the neural‐circuitry associated with heightened food‐seeking behavior that characterizes the weight loss state, and increase satiation (Rosenbaum et al. 2008). In other words, a modest 20% reduction in leptin via weight loss or statin use may be physiologically relevant, with increases in food intake, hence promoting weight gain.

The strength of our study lies in the use of primary human cells isolated from abdominal adipose tissue of lean and obese subjects. Compared to an in vivo approach, our in vitro approach allowed us to examine the direct acute effects of statins in differentiating adipocytes in the absence of concordant changes occurring in other tissues. However, our study is also limited by the in vitro design and we can only speculate about the clinical implications of decreased leptin expression on altering food intake. Furthermore, the clinical relevance of the concentrations of statins used in our in vitro experiments should be interpreted with caution (Bjorkhem‐Bergman et al. 2011). Even though we used a range of statin dosage based on several previously published in vitro experiments (Negre‐Aminou et al. 1997; Krysiak et al. 2009; Lobo et al. 2012), the plasma concentrations of statins in patients are much lower than those used in our experiment. In mitigation, it is likely that the localized concentration of statins in adipose tissue and intracellularly in adipocytes may be higher; this has not been investigated. The cellular uptake of statins is known to be mediated via active transport through several proteins. Among the active transporters known to regulate the cellular influx of statins, monocarboxylate transporter‐4 (MCT4) has been shown to be expressed in adipocytes and preadipocytes which may likely increase the intracellular concentrations of statins (Perez de Heredia et al. 2010; Petersen et al. 2017).

In summary, we show that simvastatin and atorvastatin reduce leptin expression and secretion in white adipocytes. Considering the role of leptin as an important satiety factor, the decreases in leptin may promote increased hunger and thereby may partly contribute to increased calorie intake and consequent weight gain in the long term among statin users. Our study explores the possibility of statins promoting increased food intake via decreasing leptin secretion. These effects, together with the psychological factors related to lowering of blood lipids and improved metabolic profile, could contribute to increased calorie intake. Clinically, the impact of statins on calorie intake and future weight gain should be emphasized at the beginning and this education should be maintained throughout continued statin therapy. This is important as statin treatment is being increasingly recommended for adults aged 40–75 years without a history of CVD and who have one or more CVD risk factors (Bibbins‐Domingo et al. 2016). Future studies examining the differential effect of various statins on changes in leptin, weight gain, and perceived hunger after initiation of statin therapy should also be undertaken, as well as addressing the feasibility of administering leptin or a leptin agonist together with statin therapy to preempt or mitigate any consequent weight gain.

## Conflict of Interests

VKS: Grant support – Philips Respironics Foundation (gift to Mayo Foundation); Consultant for Respicardia, ResMed, U‐Health, Rhonda Grey, Dane Garvin, Itamar, Philips Respironics, Bayer; Working with Mayo Health Solutions and their industry partners on intellectual property related to sleep and cardiovascular disease.
